# Correlation of Creatine Kinase Levels with Clinical Features and Survival in Amyotrophic Lateral Sclerosis

**DOI:** 10.3389/fneur.2017.00322

**Published:** 2017-07-03

**Authors:** Hongfei Tai, Liying Cui, Yuzhou Guan, Mingsheng Liu, Xiaoguang Li, Dongchao Shen, Dawei Li, Bo Cui, Jia Fang, Qingyun Ding, Kang Zhang, Shuangwu Liu

**Affiliations:** ^1^Department of Neurology, Peking Union Medical College Hospital, Chinese Academy of Medical Sciences, Peking Union Medical College, Beijing, China; ^2^Neuroscience Center, Chinese Academy of Medical Sciences, Beijing, China

**Keywords:** amyotrophic lateral sclerosis, creatine kinase, clinical characteristics, survival, prognosis

## Abstract

**Objective:**

To evaluate serum creatine kinase (CK) levels of amyotrophic lateral sclerosis (ALS) patients and to explore the relationship between CK levels and the clinical characteristics and survival prognosis of ALS patients.

**Methods:**

We analyzed the CK levels of 185 ALS patients who underwent long-term follow-up. The relationship between CK levels and clinical features including sex, age, disease duration, site of onset, body mass index (BMI), serum creatinine (Cr), and spontaneous electromyographic activity was analyzed by univariate analysis and multiple linear regression. Kaplan–Meier and Cox proportional hazards models were used to explore whether CK levels were independently correlated with survival prognosis of ALS.

**Results:**

Baseline serum CK was raised in 43% of participants. The median CK level was 160 U/L (range: 20–2,574 U/L), and 99% of patients had a baseline serum CK level less than 1,000 U/L. CK levels were significantly higher in male patients than in female patients [204 (169) versus 117 (111) U/L, *p* < 0.001] and in patients with limb onset ALS than with bulbar onset ALS (*p* < 0.001). CK levels were also correlated with serum Cr (*p* = 0.011) and the spontaneous potential score of electromyography (EMG) (*p* = 0.037) but not correlated with age (*p* = 0.883), disease duration (*p* = 0.116), or BMI (*p* = 0.481). Log CK was independently correlated with survival of ALS patients (HR = 0.457, 95% confidence interval 0.221–0.947, *p* = 0.035) after adjusting for age, sex, site of onset, serum Cr, and BMI.

**Conclusion:**

Serum CK levels of ALS patients were correlated with sex, site of onsite, serum Cr, and spontaneous activity in EMG. Serum CK could be an independent prognostic factor for survival of ALS patients.

## Introduction

Amyotrophic lateral sclerosis (ALS) is a fatal neurodegenerative disorder involving primarily motor neurons in the cerebral cortex, brainstem, and spinal cord that is characterized by progressive generalized muscle weakness, muscular atrophy, and upper motor neuron signs. The etiology remains unclear, and ALS is generally diagnosed based on clinical manifestations combined with an electrophysiological exam.

Several clinical laboratory tests could be abnormal in typical ALS, such as muscle enzyme creatine kinase (CK). Serum CK has been considered to be associated with muscle damage and has been used to differentiate myopathic lesions from neurogenic lesions. Since the 1970s, several studies have reported that CK could be mildly to moderately elevated in ALS patients (1–10), although mechanism of CK elevation in ALS is still not well understood. Several studies showed significantly higher CK levels in male ALS patients than in female patients (3, 4, 10) and in limb onset ALS compared to bulbar onset patients (3–5, 9, 10), but some studies did not find any difference between sexes (5, 6) and sites of onset (6). Furthermore, some prior studies found no relationship between CK levels and survival of ALS patients (3, 4, 11), although one recent study showed that low CK levels were associated with longer survival (9), and another found a positive correlation between CK levels and survival (10). Given these controversial findings, this study was conducted to describe serum CK levels in Chinese ALS patients and to explore the factors influencing CK concentration and whether it is a predictor of survival of ALS patients.

## Materials and Methods

This was a retrospective observational cohort study that included 185 ALS patients registered at Peking Union Medical College Hospital from January 2013 to December 2015. Patients fulfilled the revised El Escorial criteria (12) for clinically definite, probable or laboratory supported probable ALS were recruited. Patients with a concurrent acute infection, liver disease, or kidney disease were excluded.

The demographic features and clinical data of patients were collected, including age, sex, site of onset, disease duration, height, weight, and body mass index (BMI). Patients were followed up periodically by phone calls or face-to-face interviews, and survival status was last updated in December 2016. Survival time was calculated as the time from the date of enrollment to death or tracheotomy.

The venous blood samples of all patients were drawn after overnight fasting and rest. CK concentration was measured by a direct enzymatic method according to the International Federation of Clinical Chemists. The normal range of serum CK concentrations in our center is defined as 24–195 U/L in men and 24–170 U/L in women. Serum creatinine (Cr) was also tested in the same sample.

Electromyography (EMG) data at the time of diagnosis were also reviewed in 147 patients at our center using unique criteria. The amount of spontaneous potentials (SPs) in EMG was semiquantitatively scaled from 1 to 10 points according to the locations and intensity of the fibrillation potential, the positive sharp wave and fasciculation potentials in the tested muscles, including the sternocleidomastoid, biceps brachia, triceps brachia, extensor digitorum communis, abductor digiti minimi, abductor pollicis brevis, quadriceps femoral, tibialis anterior, gastrocnemius, and paraspinal muscles. Then, the mean SP score of all muscles was calculated for each person.

This study was approved by the Ethics Committee of Clinical Research of Peking Union Medical College Hospital (Beijing, China), and all patients signed informed consents.

### Statistical Analysis

Creatine kinase concentrations were presented as the median (quartiles) due to the non-normal distribution. The Wilcoxon/Mann–Whitney *U*-test or the Kruskal–Wallis test was used to detect the correlation between serum CK concentrations and clinical features provided as categorical variables, such as gender and site of onset. Spearman correlation analysis was used to assess quantitative variables, including age, BMI, disease duration, serum Cr, and SP score. In addition, the CK data were log transformed to generate a normal distribution. A multivariate linear regression model was used to detect any independent effect of these clinical factors on the log CK values. The Kaplan–Meier method was used to generate survival curves for patients with different CK levels (normal or raised) and compared using the log-rank test. Univariable and multivariable analyses were performed with the Cox proportional hazards (PH) model to examine the effect of baseline log CK on the survival outcome after adjusting for confounding factors. A *p*-value of <0.05 was regarded as statistically significant. Statistical analyses were performed using the SPSS 21.0 software.

## Results

A total of 185 patients (107 males, 78 females) were included in this study, and the mean (SD) age was 53 (12) years (range 20–77 years). The patients were predominantly limb onset ALS with a mean duration of 16 months from disease onset to study entry. The baseline clinical features of the participants were shown in Table S1 in Supplementary Material.

### Serum CK Levels in Patients with ALS

The median CK level for the entire group was 160 U/L (range 20–2,574 U/L), and the mean CK value was 218 U/L. Forty-three percent of patients had an abnormal CK concentration higher than the upper limit of the normal range. Ninety-nine percent of the participants had a CK level less than 1,000 U/L with only two patients with a CK level above 1,000 U/L.

### Relationship between CK Levels and Clinical Characteristics of ALS

Table 1 presents the serum CK levels of patients in different subgroups. CK levels were significantly higher in male patients than in female patients (*p* < 0.001). The percentage of male patients with raised CK levels (57/107) was also higher than that in female patients (22/78) (Pearson’s chi-square, *p* = 0.001). The CK value in upper or lower limb onset ALS was higher than in bulbar onset ALS (*p* < 0.001), but there was no significant difference between the upper and lower limb onset groups (*p* = 0.144). Univariate analysis also revealed a significant correlation between CK levels and serum Cr concentration (*r* = 0.208, *p* = 0.005), EMG SP score (*r* = 0.200, *p* = 0.015), and duration of illness (*r* = 0.168, *p* = 0.022) but no correlation with patient age (*p* = 0.883) or BMI (*p* = 0.671).

**Table 1:** Serum creatine kinase (CK) levels of patients in different gender and site of onset.

	Subgroups	*n*	Median CK (quartile, range), U/L	*p*-Value
Overall		185	160 (160, 20–2,574)	
By gender	Male	107	204 (169, 20–2,574)	<0.001
	Female	78	117 (111, 25–858)	
By site of onset	Upper limb	64	171 (152, 30–2,574)	<0.001
	Lower limb	80	197 (146, 32–1,153)	
	Bulbar	33	92 (43, 25–376)	

Multivariate linear regression model showed that log CK was independently correlated with sex (*p* = 0.001), site of onset (*p* < 0.001), serum Cr (*p* = 0.011), and EMG SP score (*p* = 0.037), but not correlated with duration of illness (*p* = 0.116) or BMI (*p* = 0.481) (Table 2).

**Table 2:** Factors significantly correlated with log CK in ALS patients by multivariate linear regression analysis.

Variables	Estimated parameter (β)	SE	95% CI	*T*	*p*-Value
Sex	0.177	0.054	0.070–0.284	3.260	0.001
Site of onset	0.254	0.063	0.129–0.378	4.033	<0.001
Serum Cr	0.005	0.002	0.001–0.009	2.580	0.011
SP score	0.025	0.012	0.002–0.049	2.110	0.037

*CI, confidence interval; Cr, creatinine; ALS, amyotrophic lateral sclerosis; CK, creatine kinase; SP, spontaneous potential*.

### Association between Serum CK and Survival in ALS Patients

**Figure 1:**
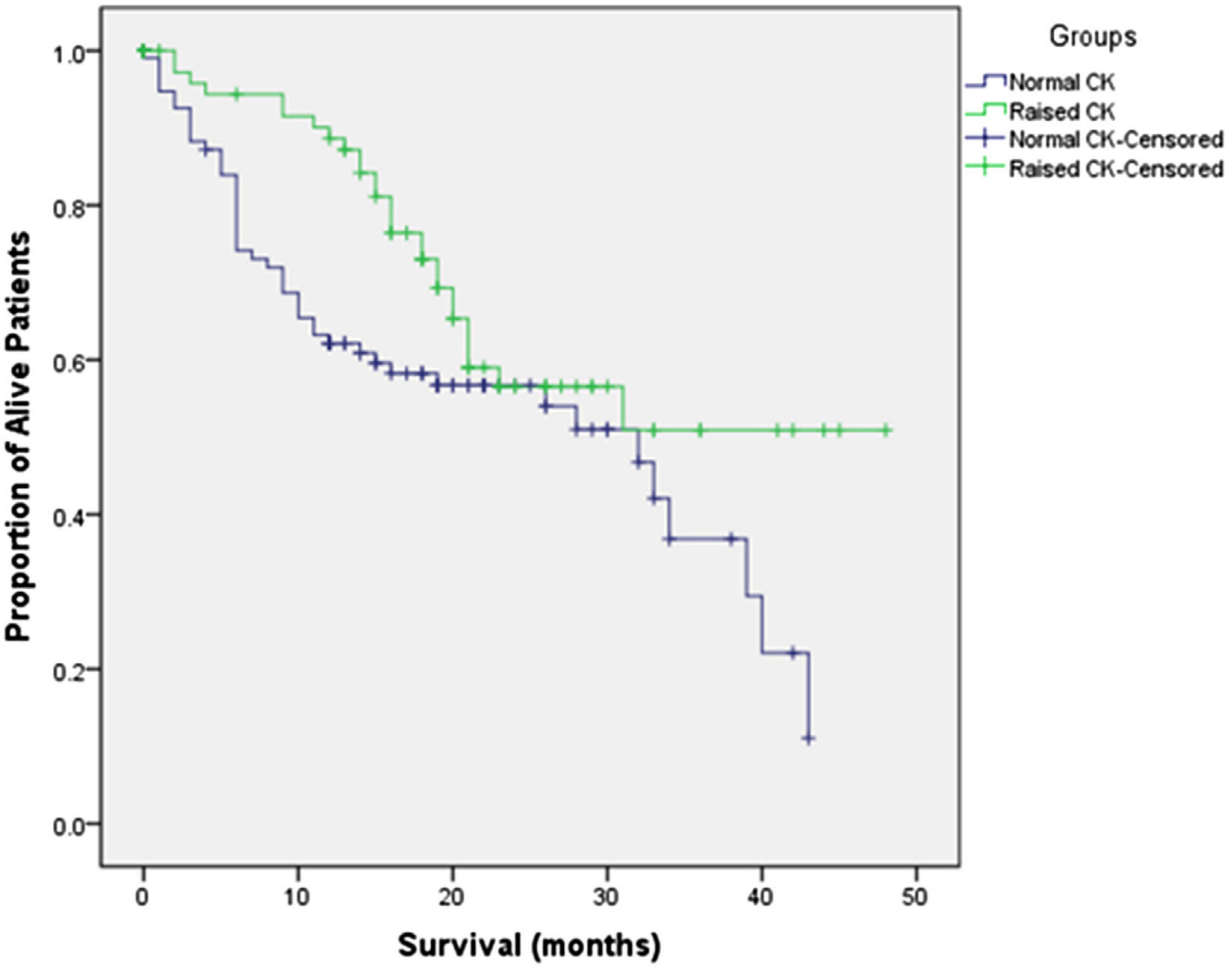
Kaplan–Meier survival curves for amyotrophic lateral sclerosis patients with normal creatine kinase (CK) versus raised CK levels.

Amyotrophic lateral sclerosis-related deaths occurred in 74 patients through December 2016. Kaplan–Meier survival curves were drawn after dividing all subjects into two groups based on normal CK and raised CK levels (Figure 1). The log-rank test indicated that patients with raised CK had significant longer survival than patients with normal CK levels (*p* = 0.030).

The Cox PH model was then utilized to examine the association between log CK and survival prognosis in ALS with or without adjusting for other covariates (Table S2 in Supplementary Material). Log CK was confirmed to be significantly associated with overall survival (Table 3). There was a 54.3% reduction in risk of death with each 1 U increase in log CK [adjusted HR = 0.457, 95% confidence interval (CI) 0.221–0.947, *p* = 0.035].

**Table 3:** Cox proportional hazards survival analysis results.

		HR (95% CI)	*p*-Value
Model I	Log CK (unadjusted)	0.347 (0.172–0.704)	0.003
Model II	Log CK (adjusted)^a^	0.457 (0.221–0.947)	0.035

*HR, hazard ratio; CK, creatine kinase*.

*^a^Adjusted for age, sex, site of onset, disease duration, serum creatinine, and body mass index*.

## Discussion

Creatine kinase is an enzyme expressed by various tissues and cell types, which catalyzes the reversible conversion of creatine and utilizes adenosine triphosphate (ATP) to create phosphocreatine and adenosine diphosphate. In tissues that consume ATP rapidly, especially skeletal muscle, CK is an important enzyme. Clinically, serum CK is assayed as a marker of CK-rich tissue damage such as in muscular disease, myocardial infarction, and acute kidney injury.

Raised serum CK concentrations are also found in ALS patients. In the present study, 43% of ALS patients had a CK concentration above the upper limit of the normal range. This is consistent with previous studies that reported 23–70% of ALS patients could have raised CK levels (2, 3, 5–10). In total, 90% of participants less than 400 U/L CK, and 99% had less than 1,000 U/L CK corresponding to all but two cases. Unlike in muscle diseases, ALS patients usually have mildly or moderately raised CK levels.

The mechanism of CK elevation in ALS has not been clearly elaborated. To date, the main hypotheses are as follows: (1) muscle energy metabolism disturbance may cause increased endogenous ATP activity in mitochondria, resulting in upregulated CK expression to provide an energy substrate (10). (2) Significantly elevated CK is associated with increased muscle cell membrane permeability due to denervated muscle and historically myopathic changes in ALS patients (13).

Several studies have shown higher serum CK levels in male ALS patients than in female patients (3, 4, 10), which is consistent with our findings. However, Gibson’s study (9) showed that the difference in CK between genders was no longer statistically significant after adjusting for bioelectric impedance spectroscopy measured fat free mass, indicating that the difference in CK values by gender could be explained by differences in muscle mass. Rafiq’s study (10) also revealed a linear correlation between serum CK and lean body mass estimated by the Boer formula. We also found a positive correlation between serum CK and Cr levels (*p* = 0.011), which is considered as a surrogate of muscle mass (14). This evidence suggests that, to a certain extent, higher CK levels might be associated with greater muscle mass. However, no studies, including the present study, directly measured the muscle mass of ALS patients using a precise instrument, which should be evaluated in future studies.

In addition, a dramatic loss of CK activity has been detected in transgenic ALS animal studies (15, 16). In G93A transgenic mice, CK activity in homogenates from spinal cords decreased to 49% and in mitochondrial fractions to 67% compared to CK activity in wild-type control mice. CK enzyme was crucial for energy metabolism and maintaining ATP levels, it might be reasonable for the compensatory upregulation of CK in this condition.

Our study confirmed that CK levels in limb onset ALS were significantly higher than those in bulbar onset ALS. It has been hypothesized that increased CK in ALS might reflect the extent of muscle involvement. As SPs in EMG recordings are a reliable sign of progressive muscle denervation, only one study has examined the association between CK levels and the number of muscles with fasciculation or fibrillation potentials in EMG recordings, although no relationship was found (6). For the first time, we developed an SP score taking into account the extent and amount of SPs in each muscle, and all muscles examined in every region and revealed that the CK value was associated with SP scores. This may support the muscle damage hypothesis to explain the raised CK mechanism. We supposed that upregulation of CK expression and muscle denervation may both have independent effects on CK levels according to the multivariable linear analysis.

There was a direct correlation between CK level and disease duration (*r* = 0.168, *p* = 0.022), but it became not significant in multivariate regression analysis (*p* = 0.116). Then we added the variables into the model step by step, the significance of “duration” turned to be unremarkable when “site of onset” was in. That is to say, the difference in CK level by duration could be explained by differences in site of onset, and no independent correlation was found between CK level and duration of illness as in prior studies (5, 10).

The influence of serum CK on survival prognosis of ALS patients has been controversial. Two prior studies did not find a significant relation between CK levels and survival of ALS patients by the Kaplan–Meier log-rank test or direct correlation analysis (3, 4). These studies might be limited due to the small sample (*n* = 30 and *n* = 73). Two additional studies conducted recently demonstrated conflicting results. One study showed that ALS patients with high baseline CK (>200 U/L) had a significantly higher hazard of death than patients with low baseline CK (≤200 U/L) after adjusting for age, gender, race, BIS fat free mass, location of onset, and study site (HR = 3.57, 95% CI 1.84–7.27, *p* < 0.05) (9). This is contrast to our results. Of note, the 68 participants in Gibson et al.’s study were recruited at five study centers with different reported upper limits of normal CK from each laboratory site (ranging from 145 to 320 U/L), but in the survival analysis, CK was considered as dichotomous variable by the same cutoff value of 200 U/L. We supposed the differences among laboratory tests and selection of the cutoff value may have influenced the study results; furthermore, the baseline disease duration was not adjusted for, which may also give rise to bias. The other study found that higher CK_log_ was significantly associated with better survival, even after adjusting for other prognostic variants (HR = 0.74, 95% CI 0.59–0.93, *p* = 0.013) (10). This report is consistent with our findings, indicating that serum CK levels are positively associated with ALS survival and could be an independent marker in ALS studies and clinical drug trials. As CK was log transmitted in the Cox PH analysis, we should cautiously interpret the result, and the effect of the original CK value on survival should not be exaggerated. On the other hand, the positive effect on survival also supported the protective mechanism hypothesis for raised CK, such as upregulated expression.

Due to the retrospective design of the study and missing data, we could not examine the relationships between CK values and the slope of ALS-FRS-R scores, forced vital capacity, and cramps. The extent and severity of cramps could also be related to CK elevation (9) and may be associated with the site of onset. Because of the short follow-up time for patients censored, the sample for the survival analysis was relatively small, and long-term observations are still needed. In addition, the association between CK and muscle mass should be confirmed with accurately tested muscle mass data. Although we suggest that some clinical features are related to CK and raised CK levels might be an overall protective factor, molecular-level studies that directly demonstrate the mechanism of CK elevation are warranted.

## Ethics Statement

This study was approved by the Ethics Committee of Clinical Research of Peking Union Medical College Hospital (Beijing, China), and all patients signed informed consents.

## Author Contributions

HT: conception of the work, data acquisition, statistical analysis, and writing of the first draft. LC: conception and organization of the work, manuscript review, and critique. YG, ML, and XL: clinical and electrophysiological evaluation of patients. DS, DL, BC, JF, QD, KZ, and SL: data acquisition, long-time follow-up of patients, and statistical analysis.

## Conflict of Interest Statement

The authors declare that the research was conducted in the absence of any commercial or financial relationships that could be construed as a potential conflict of interest.
