# Supplementary material for: Correlation of Creatine Kinase Levels with Clinical Features and Survival in Amyotrophic Lateral Sclerosis
Source: Front Neurol. 2017 Jul 3;8:322. doi: 10.3389/fneur.2017.00322 (PMC5494475; doi:10.3389/fneur.2017.00322)
Supplement: Supplementary file 1 [file Table_1.PDF]

Supplementary Table 1: Baseline characteristics of the patients (n=185)

**Variable**	n/ value
Number of patients	185
Gender (M:F)	107(58%):78 (42%)
Mean age (years) ± SD (range)	53 ± 12 (20 – 77)
Site of onset (n, %)	
Bulbar	33 (17.8%)
Upper Limb	64 (34.6)
Lower Limb	80 (43.2%)
Respiratory	3 (1.6%)
Upper + Lower Limb	4 (2.2%)
Lower Limb + Bulbar	1 (0.5%)
Mean weight (kg) ± SD (range)	64.1 ± 10.8 (30 – 90)
Mean BMI ± SD (range)	23.1 ± 3.6 (11.4 – 38.5)
Mean disease duration (months) ± SD	16.4 ± 14.3
Deceased during follow-up	
Total (%)	76 (42%)
ALS related (% of total deaths)	74 (97.4%)
Non-ALS related (% of total deaths)	2 (2.6%)
