# Supplementary material for: Correlation of Creatine Kinase Levels with Clinical Features and Survival in Amyotrophic Lateral Sclerosis
Source: Front Neurol. 2017 Jul 3;8:322. doi: 10.3389/fneur.2017.00322 (PMC5494475; doi:10.3389/fneur.2017.00322)
Supplement: Supplementary file 2 [file Table_2.PDF]

Supplementary Table 2: Univariate Cox PH model survival analysis

	P	RR
Sex	0.644	1.115 (0.702-1.771)
age	0.005	1.034 (1.010-1.058)
dura	0.001	0.955 (0.930-0.981)
Site	0.024	1.815 (1.082-3.042)
Scr	0.004	0.972 (0.953-0.991)
BMI	0.002	0.887 (0.824-0.956)
riluzole	0.635	0.848 (0.428-1.677)
logCK	0.003	0.347 (0.172-0.704)
